# Fast physical repetitive patterns generation for masking in time-delay reservoir computing

**DOI:** 10.1038/s41598-021-86150-0

**Published:** 2021-03-23

**Authors:** Apostolos Argyris, Janek Schwind, Ingo Fischer

**Affiliations:** 1 Instituto de Física Interdisciplinar y Sistemas Complejos IFISC (CSIC-UIB), Campus UIB, 07122 Palma de Mallorca, Spain; 2 Institute of Applied Physics, University of Münster, Corrensstr. 2/4, 48149 Münster, Germany

**Keywords:** Optics and photonics, Applied optics

## Abstract

Albeit the conceptual simplicity of hardware reservoir computing, the various implementation schemes that have been proposed so far still face versatile challenges. The conceptually simplest implementation uses a time delay approach, where one replaces the ensemble of nonlinear nodes with a unique nonlinear node connected to a delayed feedback loop. This simplification comes at a price in other parts of the implementation; repetitive temporal masking sequences are required to map the input information onto the diverse states of the time delay reservoir. These sequences are commonly introduced by arbitrary waveform generators which is an expensive approach when exploring ultra-fast processing speeds. Here we propose the physical generation of clock-free, sub-nanosecond repetitive patterns, with increased intra-pattern diversity and their use as masking sequences. To that end, we investigate numerically a semiconductor laser with a short optical feedback cavity, a well-studied dynamical system that provides a wide diversity of emitted signals. We focus on those operating conditions that lead to a periodic signal generation, with multiple harmonic frequency tones and sub-nanosecond limit cycle dynamics. By tuning the strength of the different frequency tones in the microwave domain, we access a variety of repetitive patterns and sample them in order to obtain the desired masking sequences. Eventually, we apply them in a time delay reservoir computing approach and test them in a nonlinear time-series prediction task. In a performance comparison with masking sequences that originate from random values, we find that only minor compromises are made while significantly reducing the instrumentation requirements of the time delay reservoir computing system.

## Introduction

Reservoir computing (RC) has been a popular computing paradigm with simplified recurrent neural network architectures^1–3^. In the various attempts to convert this paradigm into a hardware computing system, several approaches and additional simplifications have been proposed^4–7^. Among them, time delayed reservoir computing (TDRC) is a time-multiplexing approach of a nonlinear neuron coupled to a delayed feedback loop that introduces the recurrency^8–12^. The virtual nodes that are defined along the time delay provide the high-dimensional states for computing. But in order to obtain the diversity on the virtual nodes’ responses over an input signal, a masking process is applied at the input of the TDRC. This process is traditionally implemented by introducing a random sequence of values, each one of them assigned to a virtual node of the reservoir^13–20^. In principle, the diversity of the values within the sequence must be high, in order to exploit as much as possible the available dimensionality of the created state space. The masking sequence is then repeated for every time delay of the reservoir, so that every virtual node has the same connectivity with the input signal that is processed. A common way to introduce these repetitive sequences in a hardware TDRC system is by using arbitrary waveform generators; one can upload a sequence of predetermined values and generate the corresponding electrical signals with a desired repetition rate. An alternative approach that eliminates the necessity of expensive instrumentation is to generate temporal patterns from physical systems with (a) high inter-pattern diversity and (b) repeated in time. However, generating such patterns at sub-nanosecond rates is not a straightforward task. There were several reports in the past that used electronic circuits, radio-frequency photonics and laser-based systems, either to generate ultra-stable repetitive sinusoidal emission (microwave oscillators)^21–23^ or to generate non-repetitive broadband emission (chaotic oscillators)^24, 25^. But the generated signals from all these systems comply with only one of the two desired requirements; single-frequency emission could only generate a low-diversity sequence, while chaotic emission cannot seed a repetitive pattern and thus could be only used as a physical source of uncorrelated values uploaded in an arbitrary waveform generator^17^.

**Figure 1:**
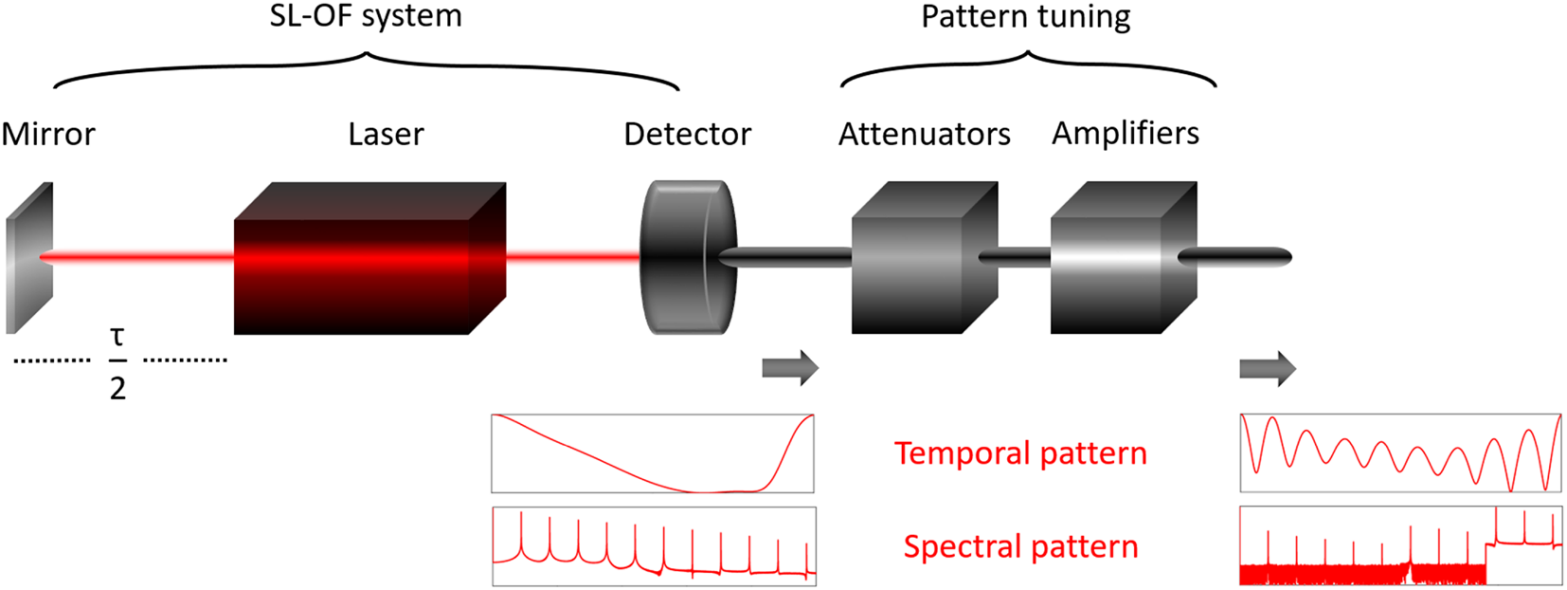
SL-OF system with external cavity round-trip time $\tau$ for generation of fast repetitive patterns with tunable intra-pattern structures. A set of microwave attenuators and amplifiers acting at different spectral regimes is used to tune the spectral profile and consequently the temporal pattern of the emitted signal.

Here we propose a very simple system which is able to generate repetitive temporal patterns with increased diversity and is based on a semiconductor laser with delayed optical feedback (SL-OF) (Fig. 1). This system has been well-explored in the last decades in terms of fundamental dynamical properties^10, 26–32^, demonstrating single-frequency^33–37^, pulsating^38, 39^ or chaotic^40–43^ emission. We explore specifically those operating conditions that result in limit cycle dynamical emission, which contains multiple harmonic frequency tones in the spectral emission profile. By tuning the relative power of these frequency tones in the microwave domain via attenuation and amplification, we generate repetitive patterns with various intra-pattern structures. In order to set an evaluation criterion regarding this diversity, we employ a permutation entropy metric that relates large variations of neighbouring samples of the pattern with high metric values. Finally, by sampling these patterns at ultra-fast rates, we generate a set of physically generated, clock-free masking sequences and test them in a TDRC one-step-ahead prediction task.

## Results

### SL with short-cavity feedback dynamics

**Figure 2:**
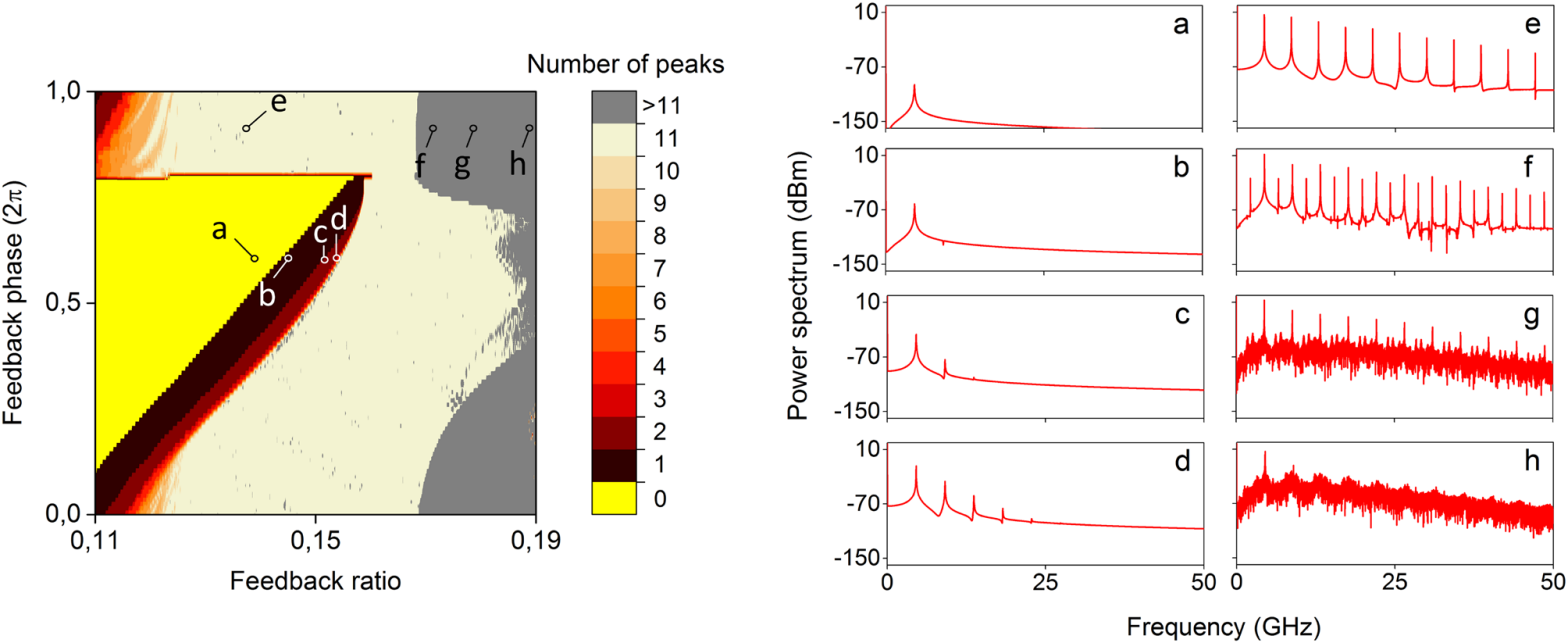
Left: Number of frequency peaks that are observed in the 50 GHz microwave spectrum of the emitted signal, versus the optical feedback ratio $r_{c}$ and phase $\phi _{c}$ of the SL-OF system. Right: Microwave spectral profiles for the different parameter conditions (**a**)–(**h**). The resolution for the parameters $r_{c}$ and $\phi _{c}$ is $10^{-4}$ and $10^{-2}$ respectively.

Semiconductor lasers with optical feedback exhibit a variety of dynamical responses, depending on their physical parameters and operating conditions^10, 26–32^. Limit cycle dynamics can be easily obtained in many parameter configurations of this system. The parameter space in which they can be observed is greatly expanded when considering short optical cavities of sub-nanosecond time delay. They may originate from single-frequency period one (P1) dynamics, but also from multiple narrow-linewidth frequency harmonic tones that appear in the microwave spectrum^44^. The latter originate from the multiple echoes of the external cavity and do not appear necessarily at the inverse delay time ($1/\tau$), but they are affected by the dynamical operation of the laser and its relaxation frequency^38, 45^. Shorter feedback cavities impose a larger spectral distance among the frequency tones. Moreover, high biasing laser current shifts the relaxation oscillation frequency to higher values, providing gain to frequency harmonics in a wider spectrum. From the numerical model of the Lang-Kobayashi equations for the SL-OF system (Methods) and in presence of laser noise, we evaluate the emitted output regarding several spectral attributes, versus the optical feedback ratio $r_{c}$ and phase $\phi _{c}$. In Fig. 2 we show the number of frequency tones that are observed in the frequency domain after photodetection (Methods) and within a bandwidth of 50 GHz. Only those peaks with power spectral density above $-80$dBm are considered, emulating a reasonable power sensitivity floor for signal detection. Zero peaks indicates an emitted signal with frequency components which are below $-80$dBm (Fig. 2, point (a), $\phi _{c} = 0.6$ and $r_{c} = 0.14$). By slightly increasing the feedback ratio for the same $\phi _{c}$ more frequency tones gain considerable power (Fig. 2, points (b)–(d)). The transition between the zones where we observe 0 and 11 frequency tones is related to the gain that these tones get under the feedback strength and phase conditions and within the operating dynamical state of the SL-OF system. The interaction between the laser emission and the re-injected optical field is strongly dependent on the phase conditions. One can observe abrupt phase matching transitions that offer gain amplification to a wide spectral region at once—and thus the 11 peaks appear almost at the same time—or phase matching transitions that offer a gradual gain increase versus frequency—and thus more tones appear gradually towards the full deployment of the 11-tone spectrum. The parameter space where we obtain 11 frequency tones within the investigated bandwidth (Fig. 2, point (e)) is rather large and is due to the combination of the high biasing current of the SL, the strong optical feedback and the short external cavity. Specifically, the relaxation oscillation frequency of the SL is high enough to provide gain to all observed peaks, while the limit-cycle dynamical operation is favoured by the short external cavity round-trip time. Even stronger feedback conditions ($r_{c}$> 0.17) combined with adjusted feedback phase may lead to more complex dynamical behavior^38, 45^, including period doubling (P2) dynamics (Fig. 2, point (f)) or coherence collapse with broadband chaotic emission (Fig. 2, points (g),(h)). Both last cases result in a larger number of frequency peaks (>11) in our mapping, represented by grey color in Fig. 2.

**Figure 3:**
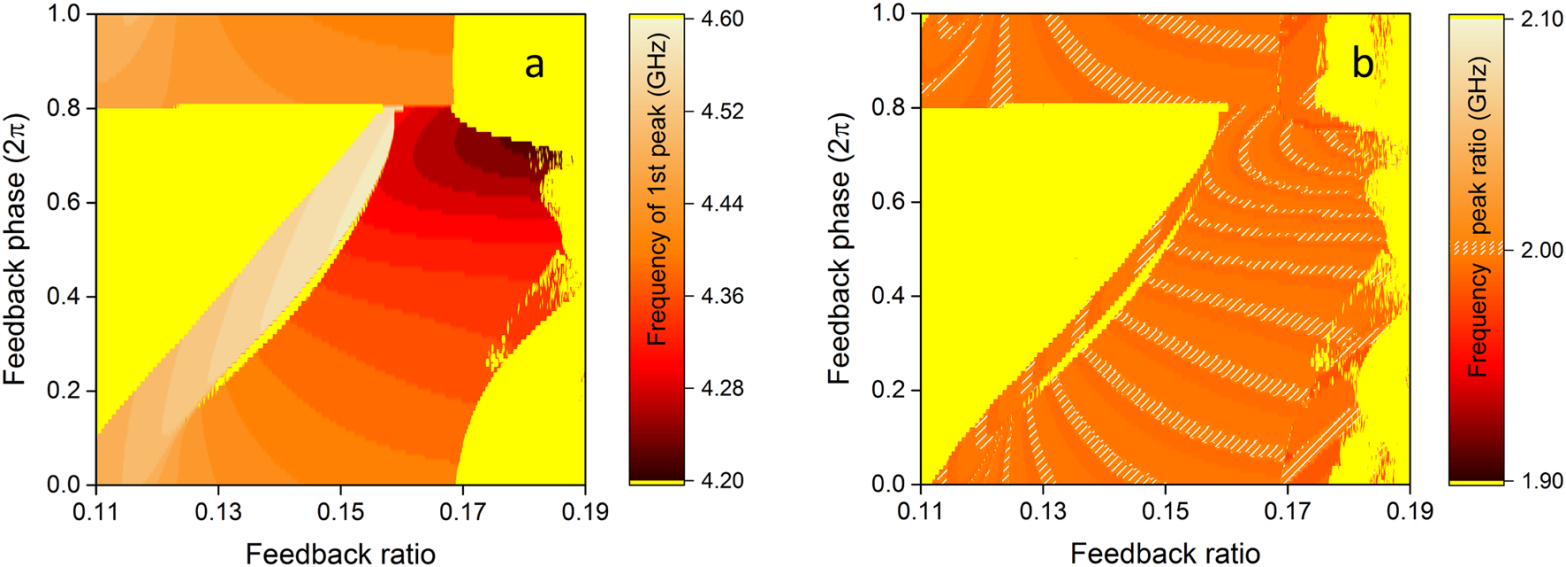
(**a**) Microwave frequency of the first tone and (**b**) microwave frequency ratio between the second and the first tone, versus the optical feedback ratio $r_{c}$ and phase $\phi _{c}$ of the SL-OF system. The white overlying lines in (**b**) highlight those conditions that satisfy an integer relation equal to 2, with an accuracy of $10^{-4}$. The resolution for the parameters $r_{c}$ and $\phi _{c}$ is $10^{-4}$ and $10^{-2}$ respectively.

Here our focus is on the dynamical response of the system with 11 multi-tone frequencies (Fig. 2, point (e)). Even though there is a large parameter tolerance to obtain this kind of emission, the attributes of the frequency tones are not the same. It has been shown in the past that tuning of the first (fundamental) frequency tone can be easily obtained by changing the feedback time delay $\tau$^46^. But even for a fixed time delay $\tau$, additional tuning can be obtained by changing the feedback conditions. This has been observed in SL-OF systems in the past with regular pulse packages^45^, where the emerging distinct spectral tones were not defined solely by the solitary laser characteristics or the external cavity round-trip time, but also by the structure of the phase space and the corresponding unstable manifolds that governed the system dynamics. In our case, this is shown in Fig. 3, where the frequency of the first tone may be tuned between 4.2 and 4.6 GHz (Fig. 3, a). Moreover, for the investigated parameter space, we have calculated the ratio between the frequencies of the second and the first tones (Fig. 3b) and we conclude that the frequency tones are not always equally spaced. Equidistant frequency tones is a necessary condition for this system to obtain repetitive patterns in the time domain. Of course, there is a more general condition under which we can obtain repetitive patterns that include multiple frequency tones in their spectrum: the great common divisor of all frequencies that appear in a pattern—if it exists—will define a periodic pattern and will give the fundamental frequency of the periodicity. However, the SL-OF system that we investigate here defines where the high-contrast frequency tones will appear in the spectrum. The frequency difference between neighbouring tones is always around the value of the frequency of the first tone. This is why we focus on the equidistance criterion and target on a periodic pattern that has as fundamental frequency the frequency of the first tone. Moreover, this guarantees the shortest possible duration of the repetitive pattern.

As shown in Fig. 3b (white highlight), there are several feedback conditions for which the frequency ratio between the second and the first tone has an integer value. For those conditions, we verify that this integer relation is also preserved between the higher frequency harmonics and the first one. Even though these highlighted regions appear quite narrow in the presented mapping, we estimate that these can be obtained experimentally in a stable operation. For example, focusing on the vicinity of a specific feedback parameter set ($r_{c}=0.136$ and $\phi _{c}=0.9$) that we will work with in this study, the equidistance criterion is preserved for $0.1343<r_{c}<0.1391$ and for $0.88<\phi _{c}<0.94$. The range of this parameter space is rather wide, even when considering an experimental implementation of this system. The feedback ratio can be tuned experimentally with large accuracy, orders of magnitude lower that the actual value. On the other hand, phase stability is always an issue in phase-dependent optical systems which are sensitive to environmental conditions. In a controlled experimental environment, the required feedback phase stability can be enhanced by active phase control mechanisms, which could in principle provide phase accuracy of less than 0.1 radians and fulfil the phase stability requirements.

### Generation of repetitive signals of increased diversity

**Figure 4:**
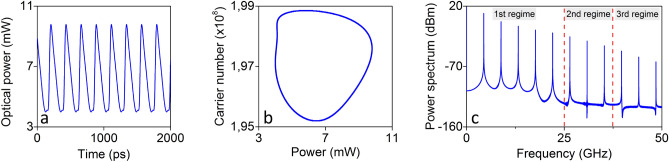
Repetitive temporal pattern emission (**a**) from a SL-OF system under a limit cycle dynamical operation (**b**) that originates from multiple equidistant microwave frequency tones (**c**). The operating conditions refer to point (**e**) of Figure 2. In (**c**) definition of the three microwave frequency regimes where we apply different attenuation / amplification conditions.

From the previous SL-OF system mapping, we select a set of feedback parameters ($r_{c} = 0.136$ and $\phi _{c} = 0.9$) (Fig. 2, point (e)) that lead to the following signal emission (Fig. 4): a quasi-sinusoidal repetitive pattern (Fig. 4a), which originates from limit cycle dynamics (Fig. 4b) and includes 11 equidistant frequency tones within a 50-GHz bandwidth (Fig. 4c). The repetition frequency of the emitted signal is $f_e = 4.4248$ GHz, equal to the frequency of the first tone. This defines a repetitive temporal pattern of duration $\tau _e=226$ ps. In this configuration, the relative power of the frequency tones gradually decreases as the frequency of the harmonics increases (Fig. 4c). This spectral signature leads to a quasi-sinusoidal pattern, with small diversity between adjacent samples (Fig. 4a). An easy way to increase this diversity is to tune the contribution of each frequency component, by using a combination of microwave attenuators and band-pass amplifiers. Here we study an even much simpler case, that requires only a limited number of components for implementation. We split the 50 GHz spectral emission into three frequency regimes: in the first regime, that covers the first 5 frequency tones up to 25 GHz, we apply microwave attenuation. In the second and the third regime, that each one includes 3 tones of higher frequencies, we apply microwave amplification (Fig. 4c). For experimental considerations there is a huge variety of microwave attenuators and amplifiers that offer attenuation or band-pass amplification gain, at the aforementioned frequency regimes.

**Figure 5:**
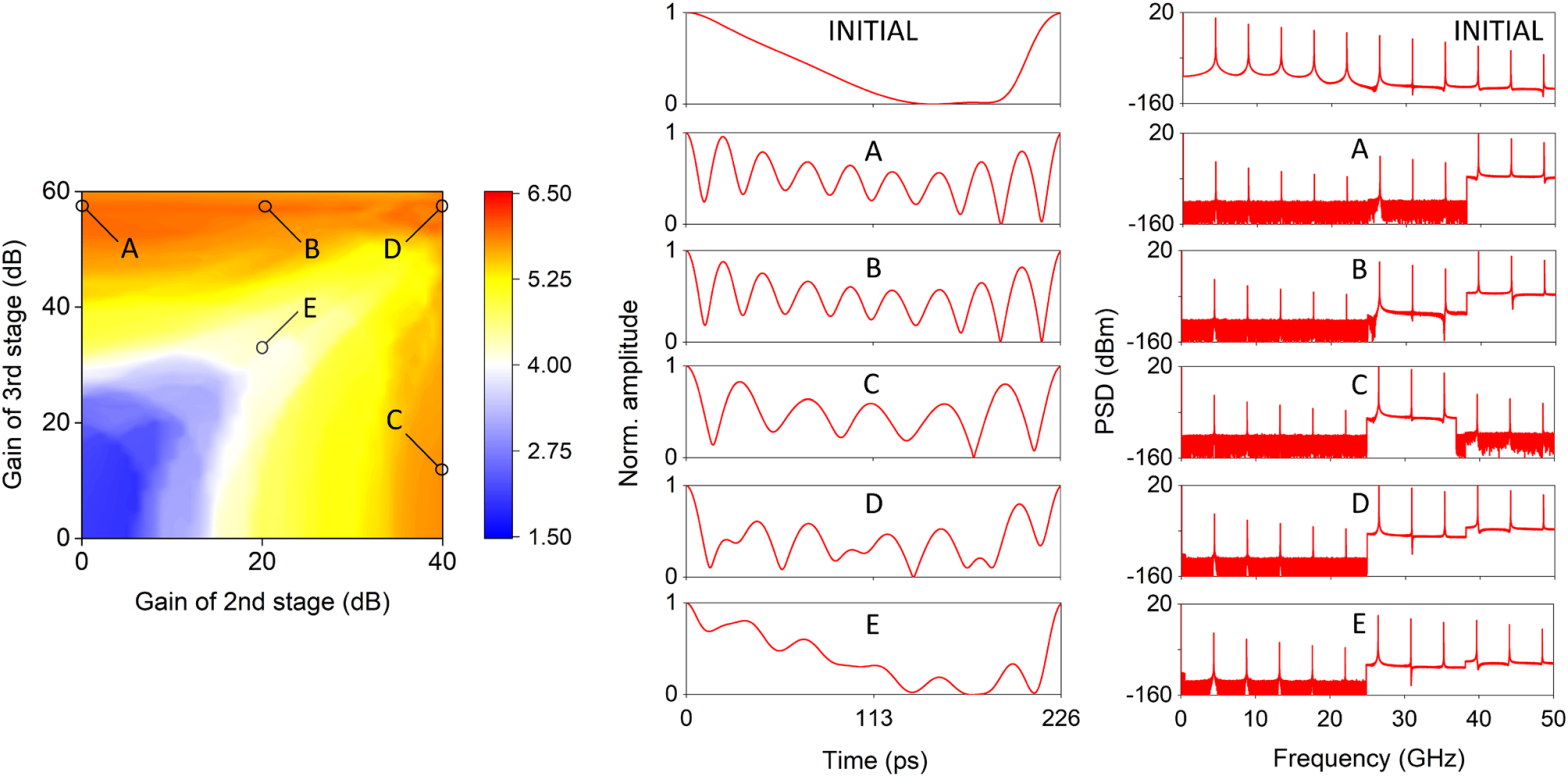
Left: Increment entropy $PE_{inc}$ of the repetitive patterns obtained for different amplification gain conditions in the second and the third spectral regime. Center: Normalized temporal persistence plots with 100 pattern repetitions for the initially emitted pattern by the SL-OF system and for the obtained patterns after electrical attenuation / amplification (cases A–E). In patterns A and B, fast oscillations are dictated by the stronger frequency components of the third amplification stage. In pattern C, the strongest frequency components of the second amplification stage result in a slower oscillating pattern. In pattern D, the strong amplification of both stages results in also faster oscillations but with suppressed peaks. In pattern E, moderate gain leads to only a limited variation of the initial pattern. The patterns are taken randomly from timeseries with 50000 consecutive patterns, including the first and the last one. The starting sample of each repetitive pattern is the one with the highest normalized amplitude. Right: Power spectral density of the corresponding patterns in a 50 GHz spectral range, as they are obtained from the physical system.

**Figure 6:**
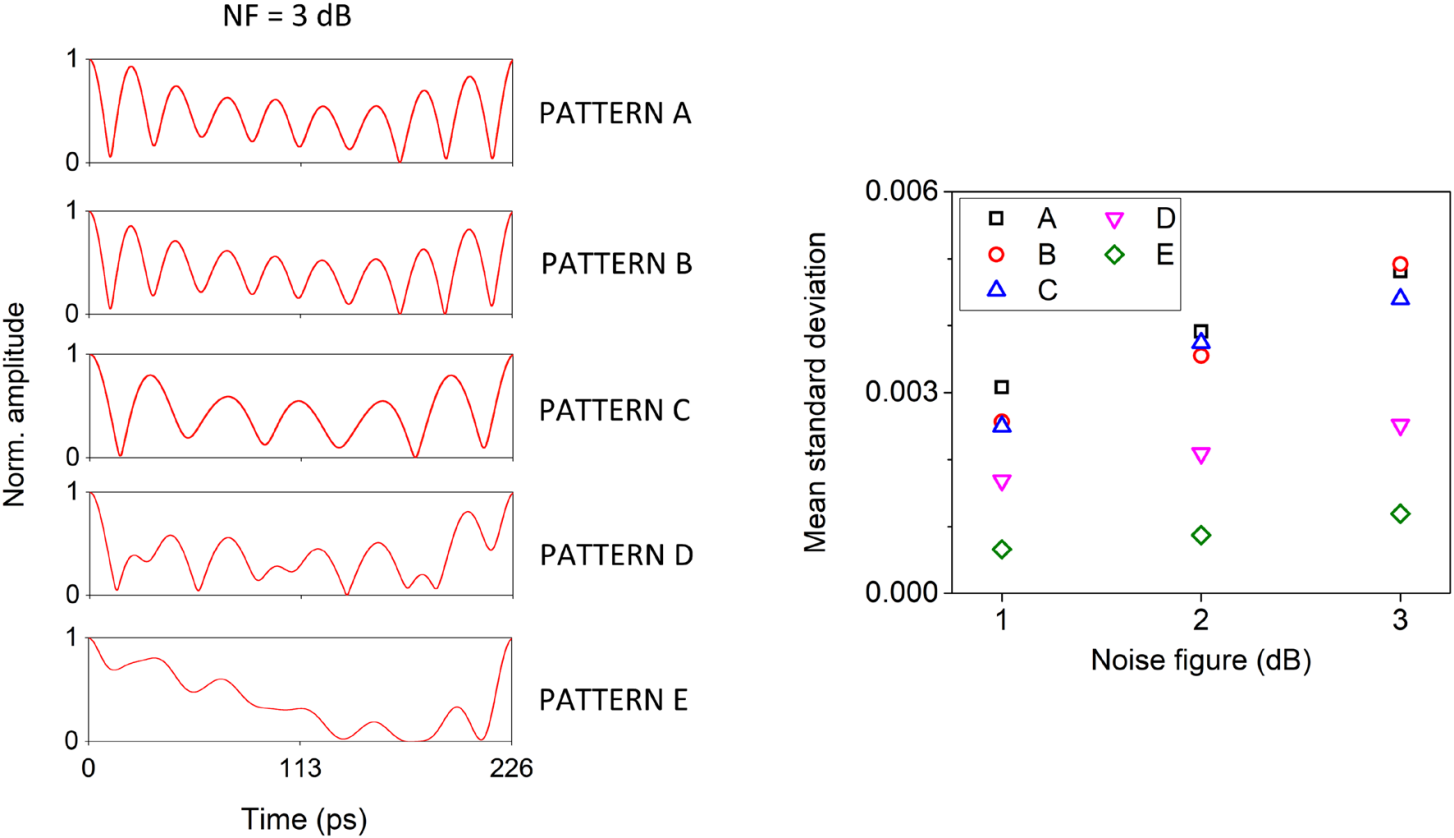
Temporal persistence plots of 100 instances of the repetitive patterns A–E, when the amplification stages suffer from *NF*=3 dB (left) and mean standard deviation of the persistence plots for different levels of *NF* (right). The temporal persistence plots are obtained from timeseries with a total duration of 50,000 patterns, including the first and the last one.

Low frequency tones contribute to the generation of slowly varying intra-pattern oscillations. Thus, we consider an attenuation of 40dB for the first stage ($G_1=-40$ dB) and suppress the most powerful, lower frequency tones. In parallel, we apply a variable amplification gain at the second ($G_2$) and the third ($G_3$) stage, in order to strengthen the contribution of the medium and high frequency tones and thus the high frequency intra-pattern oscillations. For the different amplification conditions of these two stages, we use a permutation entropy metric^47^ to evaluate the intra-pattern diversity of the generated temporal patterns. Specifically, we employ the increment permutation entropy $PE_{inc}$^48^, a modified estimator that takes additionally into consideration the absolute distance between two consecutive samples’ values (Methods). This metric can only partially describe the variability of a pattern in its total duration, since by definition it evaluates the diversity of values in the local neighbourhood of each sample. However, as we show in the next subsection, it correlates significantly with the TDRC performance. Small $PE_{inc}$ values relate to more regular patterns. Contrarily, larger $PE_{inc}$ values relate to more diverse, noisy and random patterns. Its calculation is shown in Fig. 5 (left), versus the different amplification conditions ($G_2$,$G_3$). From this, we can identify that high values of increment entropy are obtained for high gain of at least one of the amplification stages. The highest value $PE_{inc}=6$ is obtained for $G_2=0$ dB and $G_3=56$ dB (pattern A). Other high entropy conditions ($PE_{inc}\ge 5.8$) may be obtained from almost identical (pattern B) or distinct (patterns C,D) temporal patterns. Lower entropy conditions (pattern E, with $PE_{inc}=4$) come along a smaller intra-pattern variability. The temporal patterns of the illustrated cases A-E are shown in Fig. 5 (right), in a persistence plot. These plots validate that there is no drift in the periodicity, after 50000 pattern repetitions. The maximum temporal jitter of the generated patterns is measured to be less than 0.1% of the pattern duration and originates from the laser source noise. However, until now we have not considered any noise effects from the amplification stages. In a realistic scenario, such amplification stages will introduce noise; the typical noise figure (*NF*) of such components lies between 1 and 3 dB, at the frequency bands of our interest. The presence of amplification noise in the process of pattern generation is expected to have some impact on the quality of the repetitive patterns. Thus, at the next stage, we introduce an additive noise term in the pattern amplification process. The amplitude of this term is such that degrades the signal to noise ratio of the generated pattern by the *NF* value. In Fig. 6 we show the impact of introducing amplification noise to the generation process of patterns A–E. Even for the case of *NF*=3dB, the degradation of the pattern consistency is not visible from the persistence plots of the patterns (Fig. 6, left). But in order to quantify the noise effect, we calculate the mean standard deviation of each pattern from these persistence plots, for different *NF* levels (Fig. 6, right). From this graph, we conclude that the amplification noise has only a small impact on the consistency of the generating patterns, even for the case of *NF*=3 dB. This minor discrepancy might be important for applications that require pattern repetition of high fidelity, such as the TDRC.

### Masking patterns for time delay RC

In the continuation, we investigate how such physically generated patterns can be applied to TDRC for prediction tasks. We also study how the amplification noise, which enters at the generation stage of these patterns, affects the TDRC performance. The information processed by the TDRC is mapped into an expanded state space defined by the number of virtual nodes ($V_n$) of the reservoir. This mapping is implemented by multiplying each piece of input information with a repetitive masking sequence *M*, within the time duration *T* defined by the feedback loop. Each value of *M* is assigned to one virtual node ($dim(M)=V_n$) and is kept fixed throughout the computation. The operation of the TDRC and the implementation of the linear classifier at the output layer are described in Methods. The computational task in which we evaluate the generated masking sequences is a Santa Fe timeseries one-step-ahead prediction, also presented in Methods. This task has been commonly used to characterize the prediction capabilities of various reservoir computing platforms^17, 49–51^.

**Figure 7:**
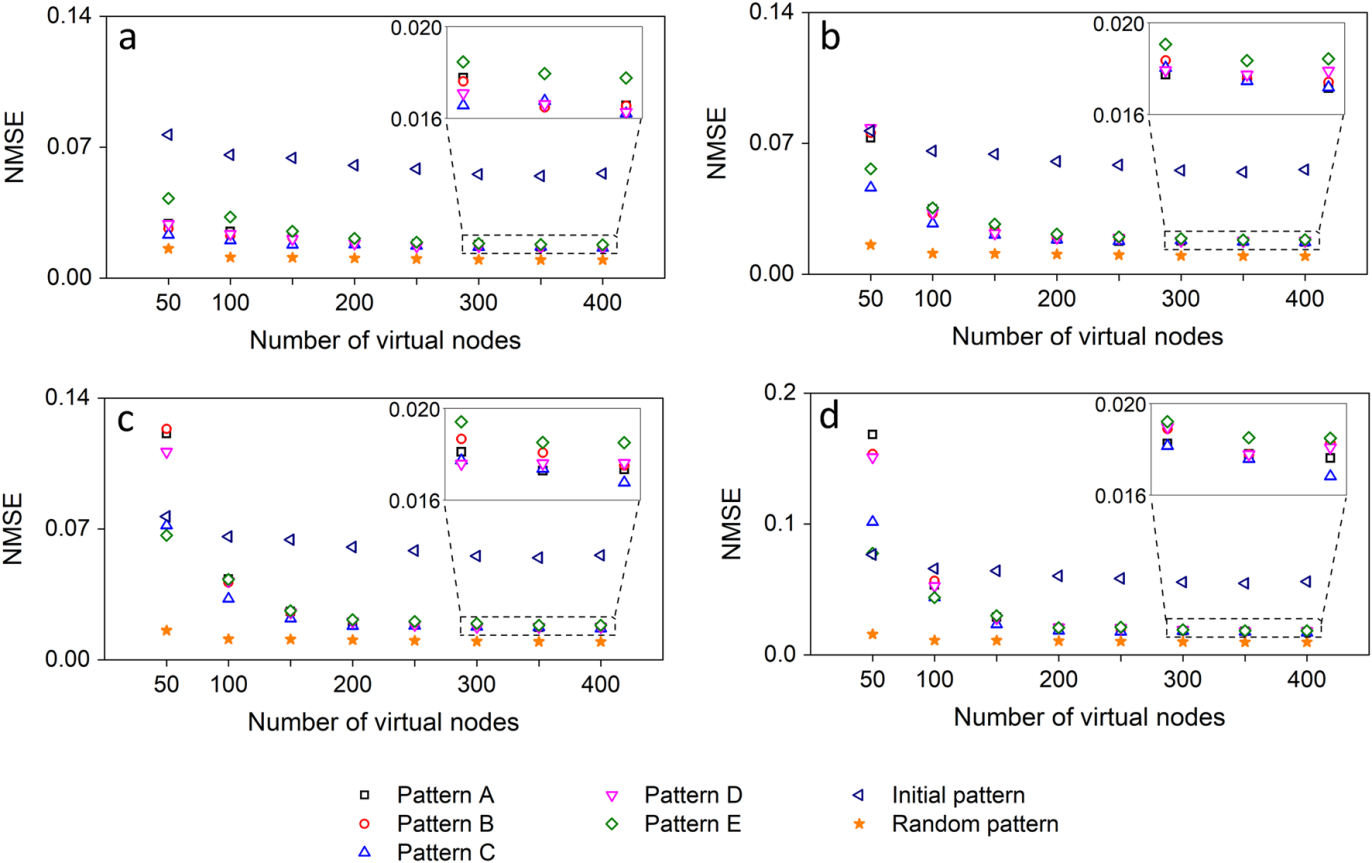
NMSE performance of the Santa-Fe timeseries one-step-ahead prediction benchmark task with TDRC, when using the sampled different patterns (A–E) that originate from the SL-OF system as masking sequences. The performance is evaluated versus the reservoir size $V_n$ and under different amplification noise conditions: (**a**) $NF=0$ dB, (**b**) $NF=1$ dB, (**c**) $NF=2$ dB and (**d**) $NF=3$ dB. The NMSE performance is compared to the one that uses as masking sequence the sampled initial pattern generated from the SL-OF system (left violet triangles). It is also compared to the one that uses as mask an algorithmic random sequence (orange stars).

The typical procedure when using arbitrary waveform generators for masking in experimental TDRC implementations is a sample-and-hold operation, where the mask value is retained for a time interval equal to the virtual node temporal spacing. In our approach, the physical generation of the repetitive patterns provides an analogue signal, with a bandwidth defined by the frequency tones and a physical duration ($\tau _e=226$ ps). This gives the flexibility to incorporate the masking sequence into a TDRC implementation in two different ways; either as a direct analogue signal or by defining a sampling rate for the pattern and following the sample-and-hold operation. In both cases, the mask sequence will be a microwave signal that will be mixed with the input data, independently of whether the TDRC refers to an electrical or an optical hardware implementation. In this study we choose to apply the masking sequences *M* to the TDRC, after sampling the physical patterns obtained from the SL-OF system. Within the very short pattern duration $\tau _e$, the number of samples that we can obtain - with large diversity between them—is limited. Thus, we sample the specific pattern duration with 50 values—since the masking sequence dimension is $dim(M)=50$—and apply it to a reservoir with $V_n=50$ virtual nodes. The sampling temporal distance with be then $\tau _e$ / $dim(M)=4.52$ ps, a value that can be monitored today by ultra-fast real-time oscilloscopes. However, in reservoir computing, the size of the reservoir is critical for the computational performance, especially in systems that include noise. Then, the question that arises is how can we use such repetitive patterns with short duration and address larger reservoirs ($V_n>50$). We circumvent this by using multiple patterns to obtain the desired masking sequence length (see also Supplementary material). For example, in order to address a reservoir with $V_n=400=50\chi$, we use $\chi =8$ repetitions of the physically sampled patterns, so that the sampling distance $(\chi \tau _e$) / $dim(M)=4.52$ ps is preserved. By following this approach, we evaluate the different masking sequences, for different reservoir sizes, in the TDRC timeseries prediction task. Specifically, we consider the originally obtained pattern from the SL-OF system and the five patterns A-E after amplification, as they are shown in Figs. 5 and 6, in absence ($NF=0$ dB) or presence ($NF=1$ dB, $NF=2$ dB and $NF=3$ dB) of amplification noise. For these patterns and noise conditions, we calculate the normalized mean square error (NMSE, see Methods) of the prediction performance in Fig. 7. We also compare their performance with the best one obtained when considering 1000 arbitrarily selected random masks, with dimension $dim(M)=V_n$ and with values drawn from a uniform normalized distribution.

The performance we obtain with the random mask (Fig. 7, orange stars) sets the floor on the lowest prediction error we can obtain. A random mask with large diversity ensures that the input information is processed while exploiting the highest possible dimensionality of the reservoir. Even with a reservoir size of $V_n=100$, the obtained NMSE reaches its minimum value, around 0.01. First, we compare this performance with the case of a masking sequence that originates from the originally obtained pattern from the SL-OF system (Fig. 5, right), in absence of any frequency tone tuning. In the illustration of the patterns in this figure, the starting sample of the repetitive pattern is the one with the highest normalized amplitude. For the TDRC task evaluation and for all the mask patterns originating from the SL-OF system, we iterate all possible shifts of the sampled mask pattern and we record the lowest error. For $V_n=50$, the NMSE obtained with a masking sequence from the initial SL-OF pattern does not get lower than 0.077, while improves to 0.055 by increasing the reservoir size to $V_n=400$ (Fig. 7, left violet triangles). However, when considering masking sequences that originate from the patterns A–E (Fig. 5, right), the NMSE lies at a significantly lower level, compared to the initial pattern. In absence of amplification noise (Fig. 7a), the patterns with high $PE_{inc}$ values result in lower NMSE. The lowest NMSE is obtained for $V_n=400$ for a masking sequence from pattern C and is equal to 0.0162. On the contrary, pattern E, which is characterized by a low $PE_{inc}$ value, is always lacking in performance. When we consider the amplification noise in the pattern generation process (Fig. 7b–d), its impact on small-sized reservoirs becomes more significant than the diversity of the masking sequence. As expected, the higher the *NF* is the stronger becomes its impact on the NMSE performance. For example, in the case of $NF=3$dB and $V_n=50$ (Fig. 7d), the error obtained from the pattern E, which is less affected by noise (Fig. 6, right), is equal to the one obtained by the initial pattern. The rest of the patterns (A–D) which are more affected by amplification noise result in higher computing error. However, the impact of noise can be dramatically suppressed by increasing the reservoir size. For $V_n=400$, the NMSE we obtain is as low as 0.0168 when considering pattern C and is almost equal to the case where amplification noise is not considered. Thus we conclude that for large reservoirs, which are more robust to the noise of the system, an increased masking sequence diversity leads to improved computing performance.

## Discussion

Although random masks result in a lower computing error compared to the ones originating from the SL-OF system, there are several arguments for overestimating their difference in performance. First, the amplification approach we selected in the presented study of the SL-OF system was towards a minimalistic design; the 11 frequency tones from the SL-OF system were clustered in three frequency bands. In an extended design, the amplitude of each frequency tone could be individually tuned. Such a consideration expands the pool of available patterns, in terms of intra-pattern diversity and thus a possibility to further optimize the performance of the computation. Second, in a real system implementation, the values defined random masking sequences must be transformed into actual electrical signals, via arbitrary waveform generators. This transformation can be only executed with finite precision in amplitude and time (jitter), while unavoidably affected by electrical noise sources. Eventually, the NMSE value obtained from a physical random mask signal is expected to be higher than the calculated value of 0.01. Third, the matching condition between the repetition period of the mask $\tau _e$ and the time delay of the reservoir *T* imposes a constraint regarding the reservoir size that we can address with a multi-tone frequency pattern. In order to apply the masking sequence to a reservoir with $V_n=400$ virtual nodes, we have repeated the same pattern $\chi =8$ times. Thus, many virtual nodes become correlated due to this pattern repetition and the extended degrees of freedom offered by the large reservoir cannot be completely exploited, as in the case of the random mask. The consideration of an even denser sampling of the patterns would not be realistic for practical implementations. However, by increasing the feedback delay time of the SL-OF system while preserving the generation of repetitive patterns, one could obtain longer masking sequences that match with the delay of larger reservoirs. The fact that we observe lower NMSE values for larger reservoirs in the proposed configuration (Fig. 7) is due to the better noise robustness of the system.

Moreover, it becomes also clear from this study that selecting an appropriate statistical metric to characterize a masking pattern and associate it to the computing performance of the TDRC is not straightforward. This appears rather reasonable since the computation performance depends also on the information that is processed. However, the increment entropy used here to quantify the diversity of the generated patterns—even though computed for neighbouring samples—is found to be significantly correlated to the task performance. The reason is that the TDRC is operated in a transient regime, so only the neighbouring virtual nodes are strongly connected through inertia. Thus, obtaining a large diversity for the neighbouring virtual nodes’ responses is much more important compared to the diversity among distant virtual nodes which are not coupled through inertia. Still, the TDRC dependence on the processing data itself, makes this metric not sufficient to implement a pre-selection of the absolute best masking pattern. For example, pattern A, which was identified from Fig. 5 with the highest increment entropy ($PE_{inc}$=6), is not always the one from which the obtained mask sequence offers the lowest computing error. But the use of this metric is very useful to discard patterns with low $PE_{inc}$ values - e.g. pattern E in Fig. 5, with $PE_{inc}$=4, or initial pattern in Fig. 5, with $PE_{inc}$=2—that result in higher computing error (see also Supplementary Fig. S4).

While TDRC topologies offer a significant reduction of complexity in the implementation of recurrent neural networks, they are limited in terms of computational speed, imposed by the time-delay of the reservoir. Thus, for masking sequences of duration $\tau _e$ that apply to a TDRC with $dim(M)=$50 virtual nodes, the computational speed that can process real-time the information with a rate $S_b$ is given by $S_b = f_e = 1/\tau _e$. A consideration to speed up the computation is the reduction of the number of virtual nodes, but with degrading the performance of the computing task. Overall, the repetitive patterns obtained from the SL-OF system offer an attractive and simple solution for the real-time, physical implementation of the masking procedure, with minimal compromises in terms of the TDRC capabilities. The pattern periodicity can be easily tuned via the feedback cavity length and the feedback parameters, while a plethora of internal pattern structures can be selected by tuning the strength of the individual frequency components. Besides the methodology we proposed here, this tuning can be implemented also by using of microwave linear filters that have appropriate spectral profiles. One could also consider to tune the frequency components in the optical domain by introducing an optical linear transformation (e.g. by employing the chromatic dispersion introduced by a fibre) and avoid any optoelectronic conversion. However, in these last considerations, the variety of patterns we one can obtain will be limited.

## Methods

### Lang–Kobayashi model for SL with optical feedback

We numerically model the SL-OF system with the Lang-Kobayashi rate equations^26^ by selecting the appropriate laser operating conditions. The slowly varying electrical field amplitude *E*(*t*) that corresponds to the optical emission of the response laser is calculated using the following equations:

1 $$\begin{aligned} {\dot{E}}(t)= & {} \frac{1}{2}(1-j\alpha )[G(t)-t_{ph}^{-1}]\cdot E(t)+\frac{r_{c}}{t_{in}}\cdot E(t-\tau )e^{j(\omega _{0}\tau +\varphi _{c})}+\sqrt{D}\xi _0 \end{aligned}$$

2 $$\begin{aligned} {\dot{N}}(t)= & {} \frac{I}{q}-\frac{N(t)}{t_{s}}-G(t)\cdot |E|^{2} \end{aligned}$$

3 $$\begin{aligned} G(t)= & {} g_{n}\cdot [1+s|E(t)|^{2}]^{-1}\cdot [N(t)-N_{0}] \end{aligned}$$

The external cavity round-trip time is set at only $\tau =200$ ps and the SL biasing current at $I=2.36 \cdot I_{th}$ (where the threshold current is $I_{th}=15.37$ mA). We consider typical values for the rest parameters of the SL: linewidth enhancement factor $\alpha =3$, gain coefficient $g_{n}=1.2 \cdot 10^{-5}\,\hbox {ns}^{-1}$, gain saturation coefficient $s=5 \cdot 10^{-7}$, carrier number at transparency $N_{0}=1.5 \cdot 10^{8}$, laser round-trip time $t_{in}=10$ ps, carrier lifetime $t_{s}=2$ns and photon lifetime $t_{ph}=2$ ps. The optical angular frequency $\omega _{0}$ is computed from the SL’s emission wavelength at 1550 nm and *q* is the electron charge. $\xi _0$ is a white Gaussian noise term with strength $D=3\,\hbox {ns}^{-2}$. This value reflects a rather low-noise laser. For a more detailed discussion on the impact of laser noise on the emitted signal properties, see Supplementary material. For the conversion of the amplitude of the electrical field to optical power^29^, we used typical values for the external and internal quantum efficiency of the laser (0.3 and 0.8 respectively). The emitted optical signal from the SL-OF system is converted to an electrical signal through photodetection. We consider photodetector responsivity of 1 A/W and no photodetection noise. In order to address a very fine temporal resolution of the generated patterns, the Lang-Kobayashi model is solved with a time step of 0.01 ps, with the 4th order Runge-Kutta approximation.

### Increment entropy

To calculate the increment entropy of the different patterns, we use the definition of^48^, which originates from the original permutation entropy definition^47^. From a pattern *P* of length *L* that is normalized in amplitude within an interval [0, 1], we form a vector with the increment values $P_i(l_u) = P(l_u)-P(l_u+1)$, with $l_u \in [1,L-1]$. Then, we use a sliding window of size $\{m\in {\mathbb {N}}\} < L$ in order to create $(L-m)$ vectors $v_k = [P_i(k),P_i(k+(m-1))]$, $k \in [1,(L-m)]$ of $P_i$. Each $v_k$ is mapped into an ordinal pattern $w_{k}$ consisting of 2*m* letters. The first *m* letters of the ordinal pattern correspond to the sign of each element of $v_k$.

4 $$\begin{aligned} w_k(q_k) = {\left\{ \begin{array}{ll} 1 &{} v_k(q_k) > 0 \\ 0 &{} v_k(q_k) = 0 \\ -1 &{} v_k(q_k) < 0 \\ \end{array}\right. }, \qquad q_k \in [1,m] \end{aligned}$$

The rest letters from $m+1$ to 2*m* get their values from the change of the amplitude of each element in $v_k$, as obtained in relation to the maximum amplitude $V_{max}$ of $v_k$, by using *R* quantization steps for each changing direction—positive or negative—to describe the amplitude change:

5 $$\begin{aligned} w_k(q_k) = \left\lceil {(|v_k(q_k)|\frac{R}{V_{max}})} \right\rceil , \qquad q_k \in [m+1,2m] \end{aligned}$$

Thus, there are $(2R+1)^m$ possible unique ordinal patterns $w_n$: R possible values for the positive change, R possible values for the negative change, and one additional value for the zero change. The increment permutation entropy results in:

6 $$\begin{aligned} PE_{inc}(R,m) = \sum _{n=1}^{(2R+1)^m}p(w_n) log_2 (p(w_n)), \end{aligned}$$

where:

7 $$\begin{aligned} p(w_n) = \frac{Q(w_n)}{L-m} \end{aligned}$$

is the relative frequency that indicates how often an ordinal pattern $w_n$ appears, with $Q(w_n)$ being the absolute frequency of its appearance. For the computation of Fig. 5, we used a four-level quantization ($R=4$), an ordinal pattern length of $m = 3$, a time delay equal to 1 (neighbouring samples). We have considered also cases where the ordinal pattern length *m* is larger (up to $m = 7$), with qualitatively identical results (see Supplementary material).

### Operation of the TDRC

In the TDRC approach, the reservoir network is constructed by a single nonlinear node and a feedback loop. It consists of:

- An input layer, which inserts the information into the reservoir. In our case the input is a vector *Y* which consists of discrete values. The connection of each piece of information with the nonlinear nodes of the reservoir is performed under fixed input weights. In time-delayed systems, where the input information is time-multiplexed along the delay of the system, these weights are assigned as a repetitive pattern sequence for every time delay. This pattern is the masking sequence (*M*).
- The reservoir, which is a nonlinear, dynamic and high-dimensional, but otherwise completely generic, network that remains unaltered at any time. The reservoir network consists of $V_n$ virtual nodes, with $V_n = T / \theta$, where $\theta$ is their equidistant time separation and *T* is the time delay of the reservoir’s feedback loop. The diversity of states is introduced by the masking sequence *M* that is repeated every time delay cycle *T*. Thus, the information to be processed *J* is multiplied with the masking sequence of dimension $dim(M)=V_n$ before being inserted in the time-delayed reservoir. *J* changes its value every time-delay of the reservoir *T*, which is also matched with the generated masking pattern duration ($T=\tau _e$). For those cases that consider not one but $\chi$ patterns repetitions ($\chi >1$, $\chi \in {\mathbb {N}}_1$) to form the masking sequence, the above condition changes to: $T=\chi \tau _e$. *M* changes its value every virtual node time separation. The recurrent connectivity via the feedback loop allows the virtual nodes to interact with their states in the past introducing fading memory. Furthermore, neighbouring virtual nodes are additionally coupled through inertia, determined by the response time of the nonlinear node.
- The output layer, where the optimal readout weights $W_{out}$ are obtained by a linear regression algorithm and the use of an appropriate training data set, for the optimal execution of the computing task.

Here we consider a generic TDRC numerical model with a *tanh* nonlinear function, a leaking rate $l_r$ and two scaling parameters $s_1$ and $s_2$, for the time-delayed and the masked input signal, respectively. The temporal response of the reservoir is solved in time steps of $\theta$ and is given by:

8 $$\begin{aligned} X(t) = l_r \cdot X(t-\theta ) + (1-l_r) \cdot \tanh \left[ s_1 \cdot X(t-T) + s_2 \cdot M(t) \cdot J(t)\right] \end{aligned}$$

where *J*(*t*) is a sample and hold representation of the input *Y*, with *J*(*t*) changing value every *T*. *M* changes every $\theta$ and repeats every *T*, while *X* is stored every $\theta$ for post-processing (see Supplementary material). We apply a scaled Tikhonov regularization to implement the classifier. We define a data subset of *n* samples of the input signal vector *Y* and for each sample we obtain the corresponding nonlinear transformation from the reservoir’s response *X* in $V_n$ dimensions. Similarly, a second data subset of *n* samples of the input signal vector *Y* and its corresponding nonlinear transformation *X* is used to test the classifier’s performance. Since our task is one-step-ahead prediction, we use $n-1$ responses of *X* to predict equal number of sample values of the input vector *Y*, but shifted by one sample. This means that we consider the training ($X_{train}$) or testing ($X_{test}$) responses of the samples 1 to $n-1$, in order to correlate them with the training ($Y_{train}$) or testing ($Y_{test}$) input vector of the samples 2 to *n*. From the reservoir responses of the training data subset ($X_{train}$) we build the matrix $M_X$, with $dim(M_X)=\{n-1,V_n\}$.

9 $$\begin{aligned} M_X = \begin{pmatrix} X_{train}^{1,1} &{} X_{train}^{2,1} &{} \dots &{} X_{train}^{n-1,1} \\ \\ X_{train}^{1,2} &{} X_{train}^{2,2} &{} \dots &{} X_{train}^{n-1,2} \\ &{}\vdots &{}\\ X_{train}^{1,V_n} &{} X_{train}^{2,V_n} &{} \dots &{} X_{train}^{n-1,V_n}\\ \end{pmatrix} \end{aligned}$$

The weight coefficients in the $\Theta$ vector with $dim(\Theta )=V_n$, are calculated from the matrix $M_X$ after row-normalization with unit variance and zero mean, denoted as ${\hat{M}}_X$. $Y_train$ is also centered to zero mean value. *k* is the regularization parameter set to $10^{-5}$ and $\mathbb {1}$ is the identity matrix.

10 $$\begin{aligned} \Theta = [{\hat{M}}_X^T {\hat{M}}_X + k \mathbb {1}]^{-1} {\hat{M}}_X^T Y_{train} \end{aligned}$$

To restore original data scale, after the normalization applied to $M_X$, we calculate the normalized weights $W_{out}^i$ of the classifier for each virtual node of index *i*:

11 $$\begin{aligned} W_{out}^i = \frac{\Theta ^i}{\sigma _{X_{train}^i}} \end{aligned}$$

where $\Theta ^i$ are the elements of vector $\Theta$ and $\sigma _{X_{train}^i}$ is the standard deviation of each row vector of the initial matrix $M_X$. The prediction values ${\widetilde{Y}}_{test}$ to the initial input vector $Y_{test}$ are computed by:

12 $$\begin{aligned} {\widetilde{Y}}_{test} = b_0 + \sum _{i=1}^{V_n}W_{out}^i\cdot X_{test}^i \end{aligned}$$

The bias term $b_0$ is calculated by introducing the mean value $\mu _{Y_{train}}$ of the input training vector $Y_{train}$ and the mean value $\mu _{X_{train}^i}$ of each row vector of the initial matrix $M_X$:

13 $$\begin{aligned}&b_0 = \mu _{Y_{train}} + \sum _{i=1}^{V_n} \mu _{X_{train}^i} W_{out}^i \end{aligned}$$

The overall task performance is evaluated via the normalized mean squared error (NMSE) between the predicted (${\widetilde{Y}}_{test}$) and the target ($Y_{test}$) vectors of the testing subset with $n-1$ samples:

14 $$\begin{aligned} NMSE = \frac{1}{n-1}\sum [Y_{test}- {\widetilde{Y}}_{test}]^2 \end{aligned}$$

### Santa Fe timeseries prediction benchmark task

The Santa Fe prediction task is based on experimental data as measured from a chaotic $NH_3$ laser system. The task is to predict a subsequent data value from the previous ones (timeseries “A” in^52^). The TDRC model for this task is optimized with parameters: $l_r = 0.4$, $s_1 = 0.6$ and $s_2 = 0.45$. In our evaluation task, the training set has a size between 600 and 3000 samples, while the testing set has a fixed size of 1000 samples. The two data sets are separated by 500 samples, in order to eliminate any correlation between the training and testing data sets. The reason for not using a fixed training set size in our classifier is to avoid any bias in cases that require larger training sets for optimized performance.

## Supplementary Information

Supplementary Information

## Data Availability

The datasets generated during and/or analysed during the current study are available from the corresponding author on reasonable request.
