# Supplementary material for: Fast physical repetitive patterns generation for masking in time-delay reservoir computing
Source: Sci Rep. 2021 Mar 23;11:6701. doi: 10.1038/s41598-021-86150-0 (PMC7988145; doi:10.1038/s41598-021-86150-0)
Supplement: Supplementary file 1 — Supplementary Information [file 41598_2021_86150_MOESM1_ESM.pdf]

Supplementary material for “Fast physical repetitive patterns generation for masking in time-delay reservoir computing”

Apostolos Argyris[1,*], Janek Schwind[1,2], and Ingo Fischer[1]

[1] Instituto de Física Interdisciplinar y Sistemas Complejos IFISC (CSIC-UIB), Campus UIB, 07122, Palma de Mallorca, Spain
[2] Institute of Applied Physics, University of Münster, Corrensstr. 2/4, 48149, Münster, Germany
*apostolos@ifisc.uib-csic.es

1. Discussion on noise effects of the SL-OF system

The presence of laser noise, as well as other noise sources and instabilities that may be present in possible physical implementations of the SL-OF system, can make it impossible to observe some of the dynamical responses of this system. For example, period-4 (P4) or even period-2 (P2) dynamics that are found numerically to exist in this system, might not be possible to observe experimentally. In this study we have considered a low-noise laser emission, which is introduced by a value of $\boldsymbol{D}$ = 3ns^{-1} in the Lang-Kobayashi model (Methods). At this laser noise level, we can observe clearly the gradual increase of the number of frequency tones (e.g. Figure 2, points (b), (c) and (d)). Moreover the fully-deployed frequency tones appear always in a wide $\{r_c,\varphi_c\}$ parameter space. When increasing the laser noise parameter $\boldsymbol{D}$ by one order of magnitude (30ns^{-1}) the gradual increase of the number of frequency tones (points (b), (c) and (d) in Figure 2) can be hardly observed. However, the parameter space $\{r_c,\varphi_c\}$ for which we observe the fully-deployed 11 frequency tones is still very wide. Regarding the conditions for which we observe the integer relation between the high order and the first frequency tones (as shown in Figure 3b), this is also significantly reduced. In this case, the feedback parameters of the SL-OF system must be defined with higher precision in order to obtain repetitive patterns without any periodicity drift.

2. Pattern repetitions for large reservoirs

In the presented analysis we select a feedback delay time of τ = 200ps and a given set of parameters for describing the laser operation. These result in the generation of repetitive patterns of τ_e = 226ps duration for the pre-selected feedback conditions. When sampling this pattern with 50 sample values, this physically defines a sampling distance of 4.52ps. Thus, for a reservoir with V_n = 50 virtual nodes, these 50 sampled values from the analogue patterns will serve as the masking values (Supplementary Figure 1). For reservoirs with larger number of virtual nodes, we use multiple patterns to reach the appropriate masking sequence length. For example, for V_n = 250, we use χ = 5 repetitions of the selected sampled pattern to obtain the desired 250 masking values.

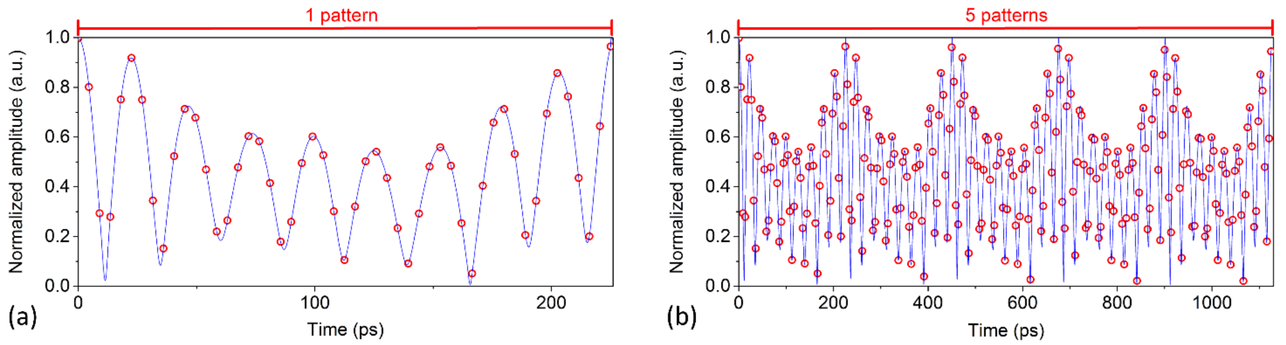

Supplementary Figure 1. Sampled values (red circles) - that coincide with the definition of the virtual nodes of the TDRC - of the analogue pattern A (blue line) of Figure 4, that form the masking sequence. **(a)** One duration of pattern-A, from which we obtain a mask sequence with 50 values, for TDRC with V_n = 50. **(b)** Five repetitions of pattern-A, from which we obtain a mask sequence with 250 values, for TDRC with V_n = 250 values.

3. Increment entropy for ordinal patterns with *m*>3

In permutation entropy calculations, longer ordinal pattern lengths consider more distant neighbouring samples. In this case, the generated multi-dimensional vectors are mapped into a larger number of unique permutations. In Figure 5 we presented the PE_{inc} metric computation with m=3 and R=4. In this case, there are $(2R+1)^m$ = 729 possible unique ordinal patterns that are considered. This number increases exponentially when increasing the value of m. Even though the highest value of the PE_{inc} metric increases slightly with the ordinal pattern length, the qualitative interpretation of our results remains the same (Supplementary Figure 2). The conclusion that high PE_{inc} values can be obtained from the repetitive patterns of the SL-OF system when at least one of the 2nd and 3rd amplification stages has significant gain is still valid for m>3.

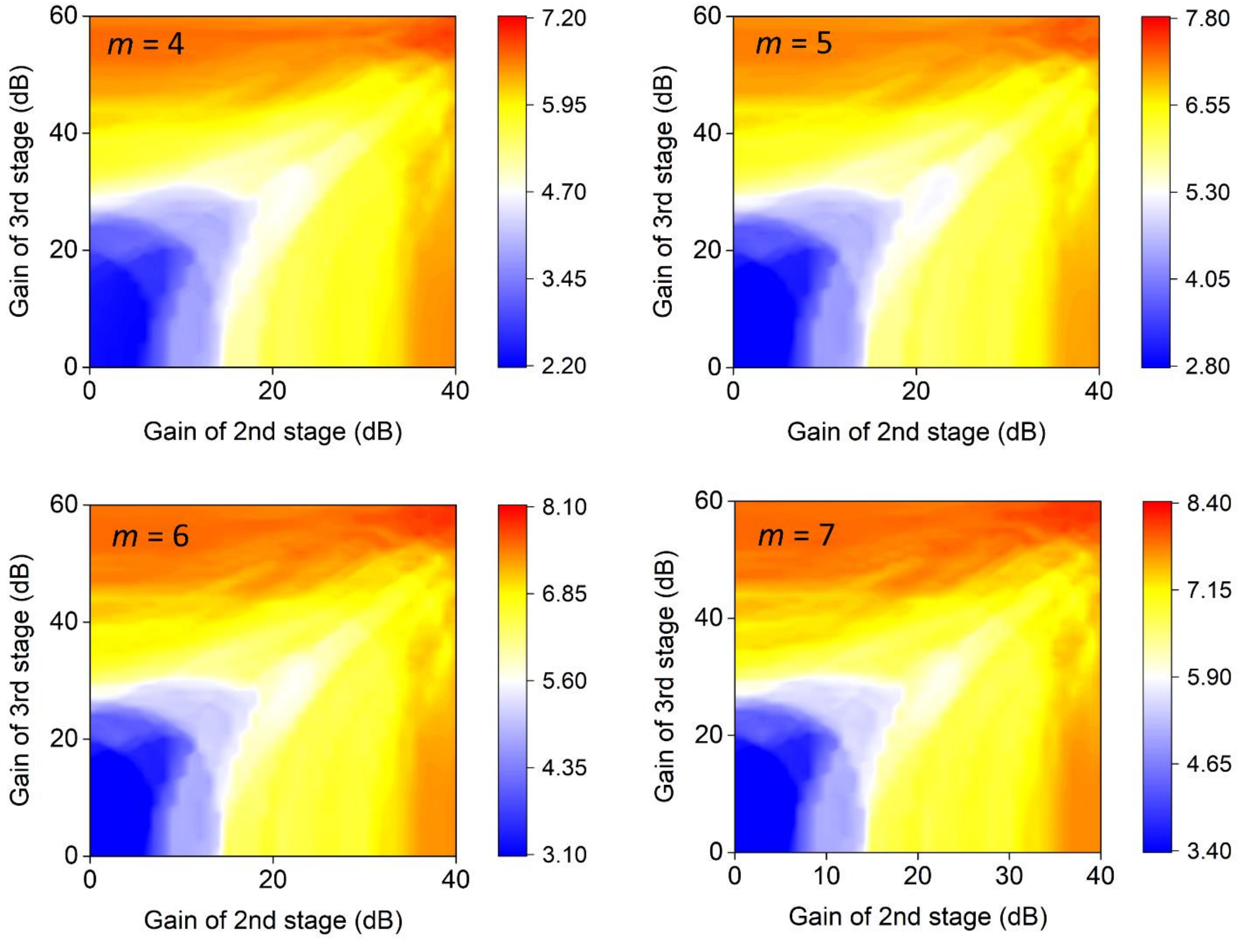

Supplementary Figure 2. Increment entropy PE_{inc} of the repetitive patterns obtained for different amplification gain conditions at the second and the third spectral regime, when considering an increased length of ordinal patterns, from m=4 to m=7.

4. Masked input and time delay reservoir response

While the response of the time delay reservoir is computed for every time step t, as given by equation (8), we only use the specific samples which are allocated to the virtual nodes of the reservoir and create a matrix representation for implementing the input layer of the TDRC. The schematic of Supplementary Figure 3 shows how the masking sequence and the input are applied in the operation of the TDRC. The masking sequence M – obtained from the repetitive patterns of the SL-OF system - consists of V_n values, with temporal distance θ and has a repetitive temporal duration of T. A change of the masking value is applied only after a temporal duration of θ, under a sample and hold operation. The input Y with dimension n x 1, with n being the total number of samples, changes its value only after every temporal duration of T. From equation (8) we obtain the temporal nonlinear transformation of the masked input, from which we retain the reservoir's responses X, in a form of matrix with dimension n x V_n, to the input samples Y. Thus, in the representation showed in

Supplementary Figure 3, the first input sample ***Y(1)*** is expanded - after the masking and the reservoir transformation - into the vector ***X(1)*** with V_n values. From the trained classifier and the defined weights, a prediction value ***Ỹ(2)*** is calculated from the ***X(1)*** and compared to the initial input value ***Y(2)***. Eventually, the first ***n-1*** responses of ***X*** are used to make the prediction ***Ỹ***, shifted by one sample.

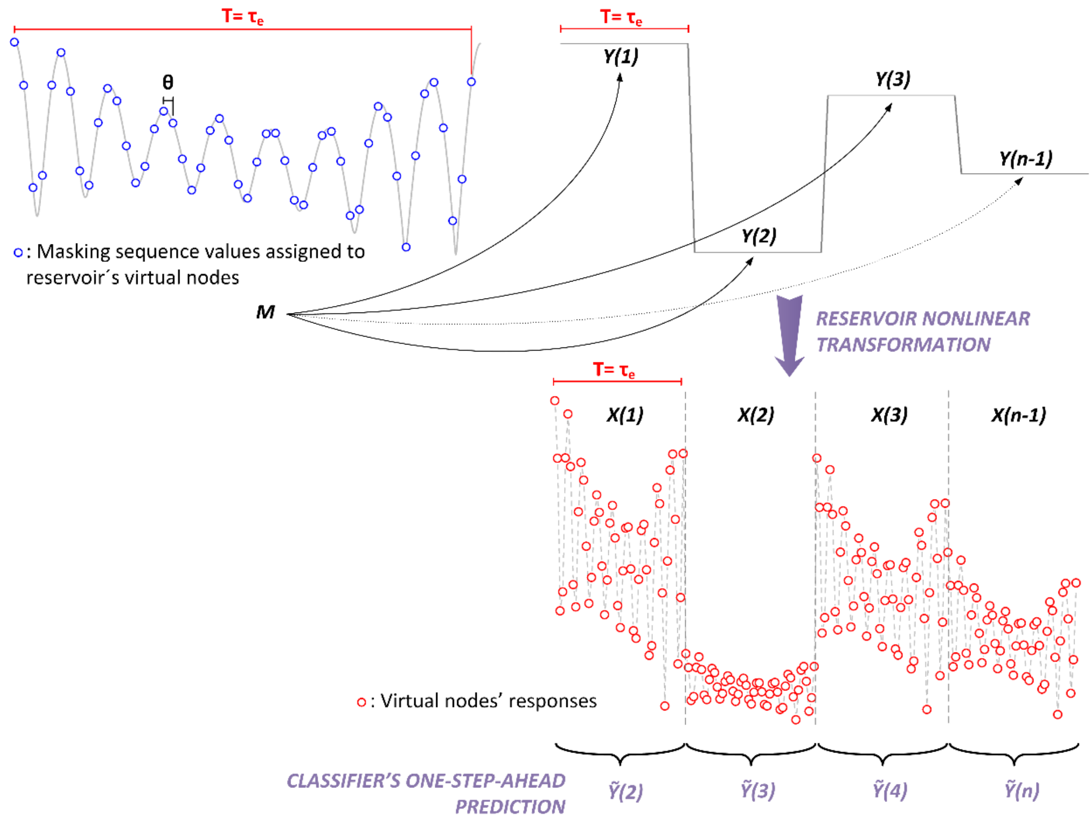

Supplementary Figure 3. Masking methodology and reservoir operation. The input sequence ***Y*** is masked by the repetitive pattern ***M*** and it goes through a nonlinear transformation by the time delay reservoir, resulting in the response ***X***. These responses are used to calculate the classifier’s one-step-ahead prediction value of ***Ỹ***.

5. NMSE performance versus PE_{inc}

Another approach to visualize the NMSE performance of the computational task for the different masking sequences, as presented in Figure 7, is versus the increment entropy (PE_{inc}) of the evaluated pattern. The connection between the patterns’ profile and their corresponding PE_{inc} value can be easily extracted from Figure 5. Here we associate the pattern ID, with the calculated PE_{inc} value, as extracted from Figure 5:

**Pattern ID**	**PE_{inc} value**
Initial pattern from SL-OF system	2
Pattern A	6
Pattern B	5.9
Pattern C	5.8
Pattern D	5.9
Pattern E	4

In a new visualization of the presented results in Figure 7, we show in Supplementary Figure 4 the NMSE performance of the different patterns A-E, including also the initial pattern from the SL-OF system, versus their PE_{inc} value. In order to keep a clear visualization, we only show here the NMSE performance for V_n=50 and V_n=400, for all the investigated NF conditions (Supplementary Figure 4, a -d) of the amplification stages.

Focusing first on the case where no amplification noise is considered (Supplementary Figure 4, a), the use of the initial pattern from the SL-OF system as masking sequence with a much lower $\boldsymbol{PE_{inc}}$=2, results in much higher NMSE for the computing task. Especially for large reservoirs ($\boldsymbol{V_n}$=400) where lower error values are obtained, the masking patterns with high $\boldsymbol{PE_{inc}}$ (≥5.8) always provide lower errors compared to the pattern with $\boldsymbol{PE_{inc}}$=4. However, this relation of NMSE versus $\boldsymbol{PE_{inc}}$ is not always linear when we zoom at the region of high entropy patterns (≥5.8); for example, there are cases, such as in Supplementary Figure 4, a and d, that the pattern C exhibits lower NMSE error than the pattern A. The discussion on the impact of amplification noise, versus the reservoir size and the masking pattern properties is included in the manuscript.

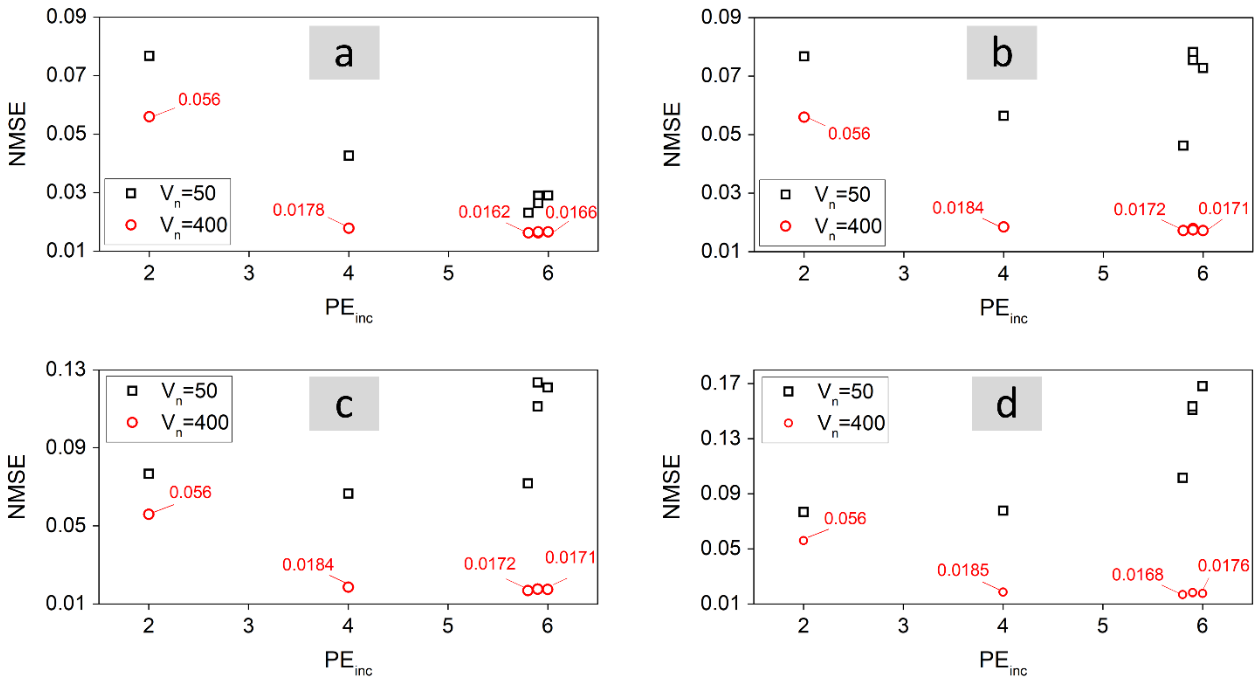

Supplementary Figure 4. NMSE performance of the Santa-Fe timeseries one-step-ahead prediction benchmark task with TDRC versus the increment entropy $\boldsymbol{PE_{inc}}$ of the different evaluated patterns, as they appear in Figure 5, after their sampling to masking sequences. The NMSE performance is shown here versus two reservoir sizes ($\boldsymbol{V_n}$=50 and $\boldsymbol{V_n}$=400) and under different amplification noise conditions: (a) $\boldsymbol{NF}$=0dB, (b) $\boldsymbol{NF}$=1dB, (c) $\boldsymbol{NF}$=2dB and (d) $\boldsymbol{NF}$=3dB.
